# Author Correction: Assessing global drinking water potential from electricity-free solar water evaporation device

**DOI:** 10.1038/s41467-024-52275-9

**Published:** 2024-09-05

**Authors:** Wei Zhang, Yongzhe Chen, Qinghua Ji, Yuying Fan, Gong Zhang, Xi Lu, Chengzhi Hu, Huijuan Liu, Jiuhui Qu

**Affiliations:** 1 Center for Water and Ecology, State Key Joint Laboratory of Environment Simulation and Pollution Control, School of Environment, Tsinghua University, Beijing, China; 2 Key Laboratory of Drinking Water Science and Technology, Research Center for Eco-Environmental Sciences, Chinese Academy of Sciences, Beijing, China; 3 University of Chinese Academy of Sciences, Beijing, China; 4 Department of Geography, The University of Hong Kong, Hong Kong, China; 5 School of Environment, Northeast Normal University, Changchun, China

**Keywords:** Environmental sciences, Sustainability

Correction to: *Nature Communications* 10.1038/s41467-024-51115-0, published online 8 August 2024

In this article the scale bar in Fig. 4 was omitted. The original article has been corrected.

