# Supplementary material for: Assessing global drinking water potential from electricity-free solar water evaporation device
Source: Nat Commun. 2024 Aug 8;15:6784. doi: 10.1038/s41467-024-51115-0 (PMC11310480; doi:10.1038/s41467-024-51115-0)
Supplement: Supplementary file 1 — Supplementary information [file 41467_2024_51115_MOESM1_ESM.pdf]

Supplementary information

Assessing Global Drinking Water Potential from Electricity-free Solar Water Evaporation Device

Wei Zhang [1, 2, 3], Yongzhe Chen [4], Qinghua Ji [1*], Yuying Fan [2, 5], Gong Zhang [1], Xi Lu [1], Chengzhi Hu [2, 3], Huijuan Liu [1], and Jiuhui Qu [1, 2, 3 *]

[1] Center for Water and Ecology, State Key Joint Laboratory of Environment Simulation and Pollution Control, School of Environment, Tsinghua University, Beijing 100084

[2] Key Laboratory of Drinking Water Science and Technology, Research Center for Eco-Environmental Sciences, Chinese Academy of Sciences, Beijing 100085, China

[3] University of Chinese Academy of Sciences, Beijing 100049, China

[4] Department of Geography, The University of Hong Kong, Hong Kong, China

[5] School of Environment, Northeast Normal University, Changchun 130117, China

Contents:

Supplementary Note 1. Fabrication of the coating of the glass 4
Supplementary Fig. 1 Distribution of the population without safely managed drinking water (SMDW) according to the annual precipitation. 4
Supplementary Fig. 2 Distribution of the population without safely managed drinking water (SMDW) according to the income levels. 5
Supplementary Fig. 3 The picture of the setups of the case 1–5. 5
Supplementary Fig. 4 The conductivity of the produced water by solar water evaporation (SWE). 6
Supplementary Fig. 5 The ion concentration of the produced water by solar water evaporation (SWE). (a) Cl^-. (b)SO_4^{2-} (The red dashed line is the World Health Organization-defined criteria). 7
Supplementary Fig. 6 Operation parameters in cases 1–5 and of the environment. 8
Supplementary Note 2. The dependence of solar-water energy efficiency 9
Supplementary Fig. 7 The linear regression between solar-water energy efficiency and (a) Day (Slope=-0.03, *p*=0.51, ns), (b) Solar irradiance (Slope=3.6, *p*=0.001, ***). (c) Between-group comparisons of the day, energy efficiency, and solar irradiance (Day vs. Energy efficiency, *p*>0.05, ns; Energy efficiency vs. Solar irradiance, *p*<0.0001, ****). 9
Supplementary Table 1 The Spearman coefficients between solar-water energy efficiency and Day or Solar irradiance 10
Supplementary Fig. 8 Data processing workflow of the physics-guided machine learning model. 11
Supplementary Fig. 9 The selected sites for the training sets of the physics-guided machine learning model. 12
Supplementary Table 2. Locations of the selected 30 cities over the world 12
Supplementary Fig. 10 The importance of each input parameter in the physics-guided machine learning model for (a) evaporation-optimized case (Eva. opt.) and (b) evaporation-condensation-optimized case (Eva.-cond. opt.). 13
Supplementary Fig. 11 Global distribution of the surface downward shortwave irradiation (DSW). 14
Supplementary Fig. 12 The violin plot of annual average safely managed drinking water (SMDW) yield under the three scenarios. 14
Supplementary Fig. 13 Seasonal variations of the safely managed drinking water (SMDW) yield for 6 six representative cities across the world. 15
Supplementary Fig. 14 Pyramid chart of country data on average annual water production and water-scarce population (evaporation-condensation-optimized system). 16
Supplementary Fig. 15 Four quadrant charts of the annual safely managed drinking water (SMDW) yield (evaporation-optimized system) concerning the population without SMDW of different income-level countries. 17
Supplementary Fig. 16 Pyramid chart of country data on average annual safely managed drinking water

(SMDW) production and water-scarce population (evaporation-optimized system). 18
Supplementary Fig. 17 Four quadrant charts of the annual safely managed drinking water (SMDW) yield (Upper limit) concerning the population without SMDW of different income-level countries. 19
Supplementary Fig. 18 Pyramid chart of country data on average annual safely managed drinking water (SMDW) production and water-scarce population (upper limit) 20
Supplementary Fig. 19 Map of the Capital expense per capita of SWE (evaporation-condensation-optimized system). 21
Supplementary Fig. 20 Schematic diagrams of the COMSOL model setups. 22
Supplementary Note 3. Cost estimation 22
Supplementary Table 3 The capital cost per area 22
Supplementary Table 4 Labor cost estimation 24

Supplementary Note 1. Fabrication of the coating of the glass

Typically, sodium alginate (SA) and polyvinyl alcohol (PVA) was dissolved by the mass fraction of 4 wt% and 10 wt%, respectively. SA and PVA solutions were mixed by a ratio of 2:1 (v/v). A glass plate was subsequently cleaned with acetone, ethanol, and water. Then this glass plate was immersed in the PEI aqueous solution (10 wt%) for 30 s and taken out to be dried at room temperature. Syringe nozzles with holes (1.0 mm in diameter) in a row were fabricated by 3D printing, and the spacing between aligned holes was designed at 1.0 mm. Before coating the HWT onto the glass plate, the glass plate was frozen to 263.15 K in the refrigerator. A syringe pump was used to control the injection rate of the HWT precursor (1.5 mL min^{-1}), and the scan rate of the syringe nozzle was set as 5 mm s^{-1}. After coating, the precursor quickly got frozen, and the glass plate was stored in the refrigerator at 253.15 K for 2 h. Then it was taken out to thaw and this freeze-thaw process was repeated 3 times. Then it was immersed in 0.25 mol L^{-1} $CaCl_2$ solution for 30 min and then taken out for rinsing.

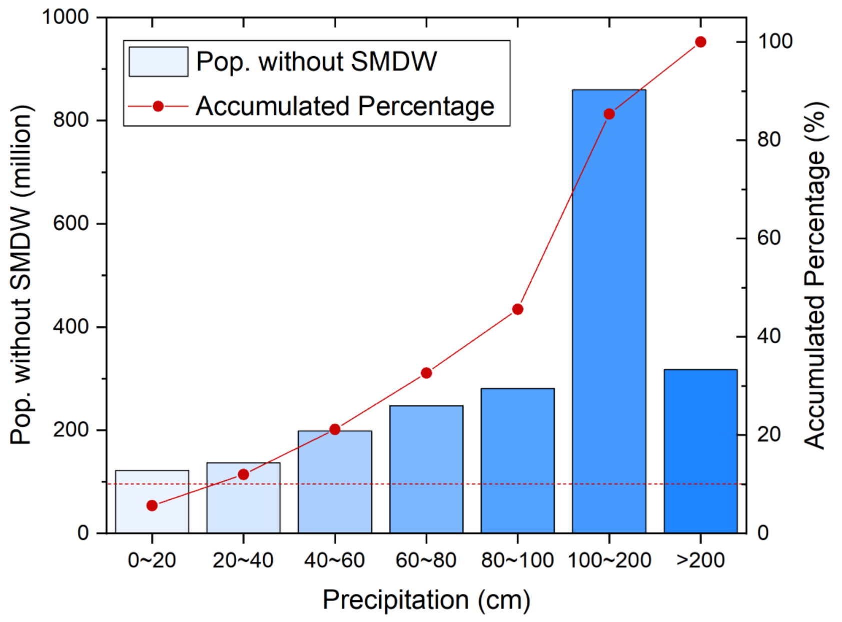

Supplementary Fig. 1 Distribution of the population without safely managed drinking water (SMDW) according to the annual precipitation.

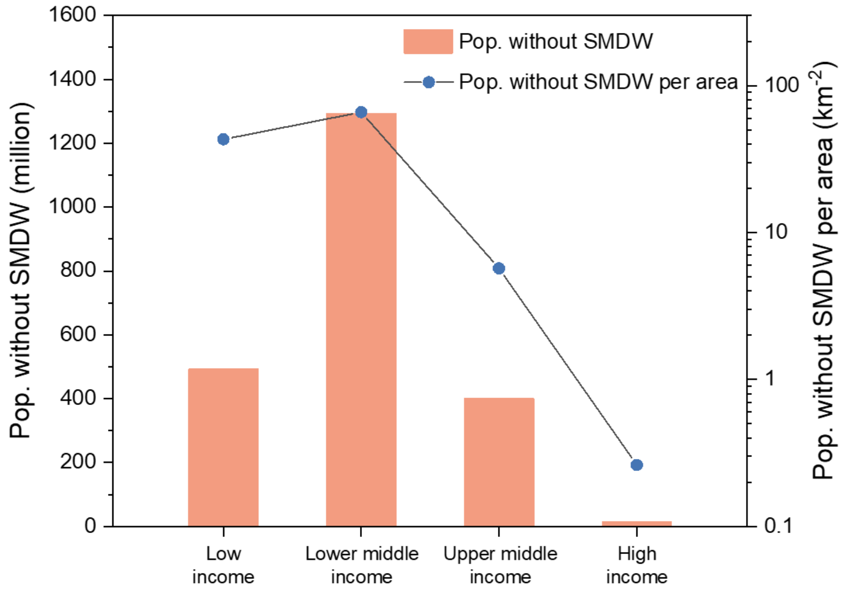

Supplementary Fig. 2 Distribution of the population without safely managed drinking water (SMDW) according to the income levels.

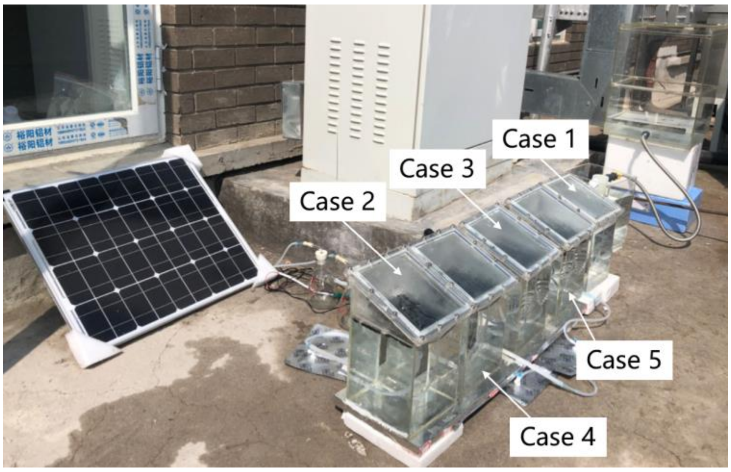

Supplementary Fig. 3 The picture of the setups of the case 1–5. Case 1 is a reference system without solar evaporators. Case 2 includes solar evaporators. Case 3 further pumps vapor out through a condensing tube for forced condensation with additional photovoltaics. Case 4 uses coated glass (condensation-enhanced) to condense the water without external energy input. Case 5 integrates both the condensing tube (powered by photovoltaics) and condensation-enhanced glass for condensation.

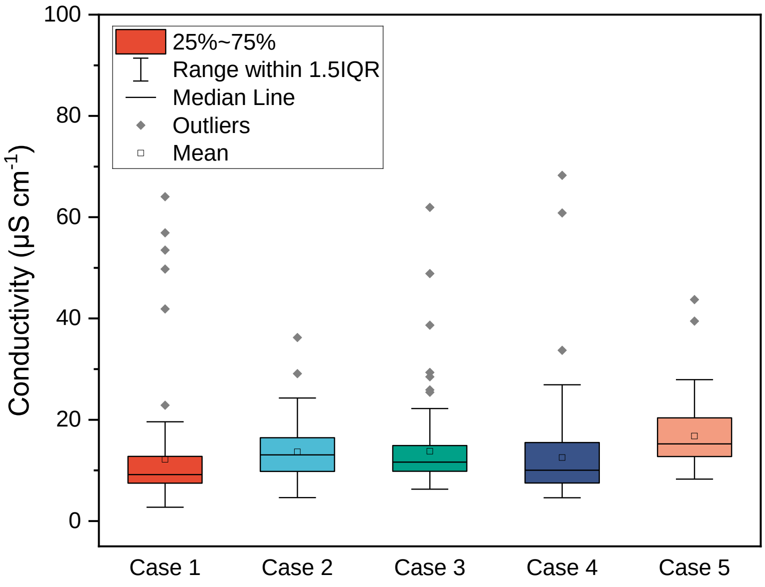

Supplementary Fig. 4 The conductivity of the produced water by solar water evaporation (SWE). Interquartile Range, IQR. Case 1 is a reference system without solar evaporators. Case 2 includes solar evaporators. Case 3 further pumps vapor out through a condensing tube for forced condensation with additional photovoltaics. Case 4 uses coated glass (condensation-enhanced) to condense the water without external energy input. Case 5 integrates both the condensing tube (powered by photovoltaics) and condensation-enhanced glass for condensation.

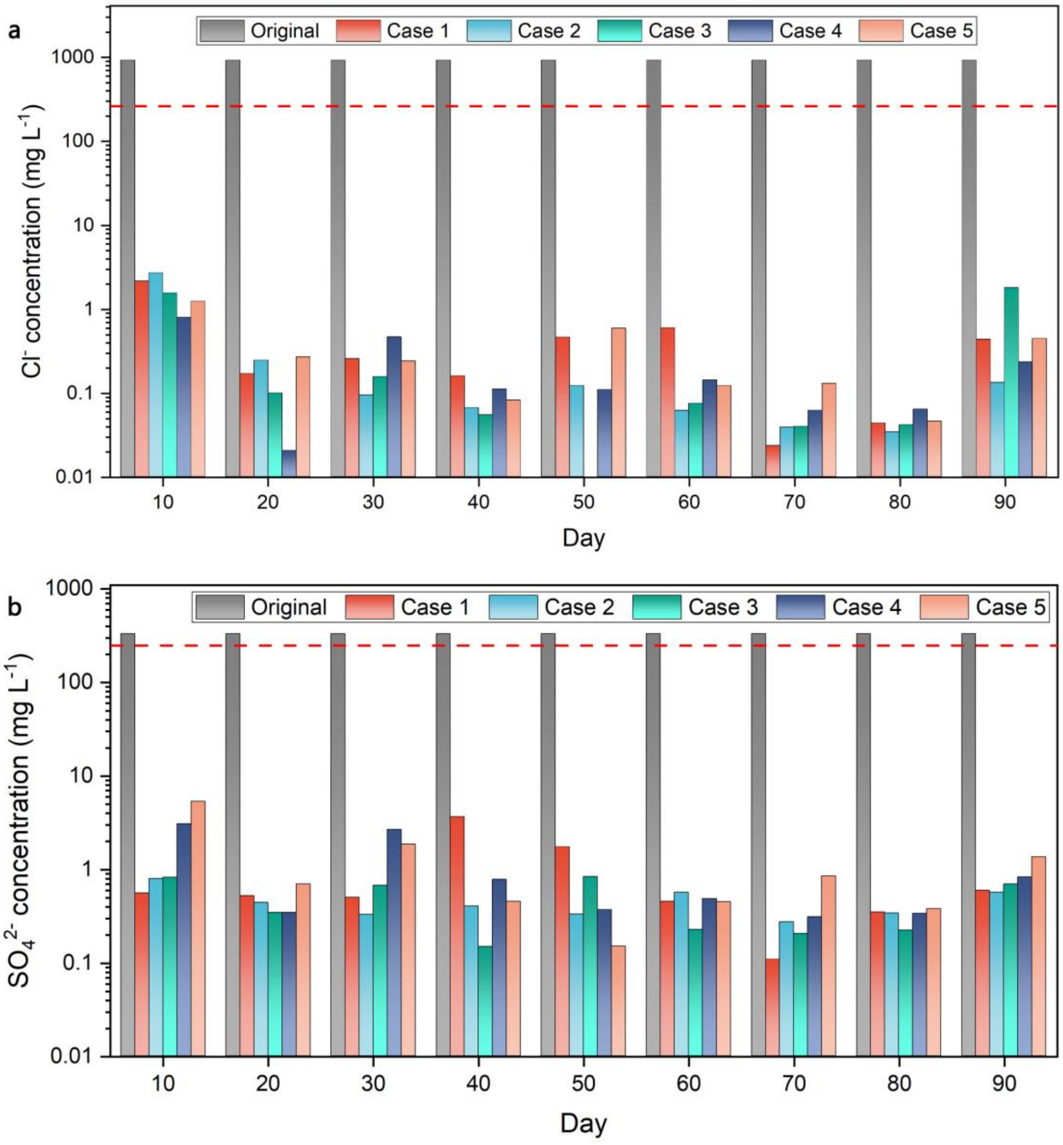

Supplementary Fig. 5 The ion concentration of the produced water by solar water evaporation (SWE). (a) Cl^-. (b) SO_4^{2-}. The red dashed line refers to the World Health Organization-defined criteria. Case 1 is a reference system without solar evaporators. Case 2 includes solar evaporators. Case 3 further pumps vapor out through a condensing tube for forced condensation with additional photovoltaics. Case 4 uses coated glass (condensation-enhanced) to condense the water without external energy input. Case 5 integrates both the condensing tube (powered by photovoltaics) and condensation-enhanced glass for condensation.

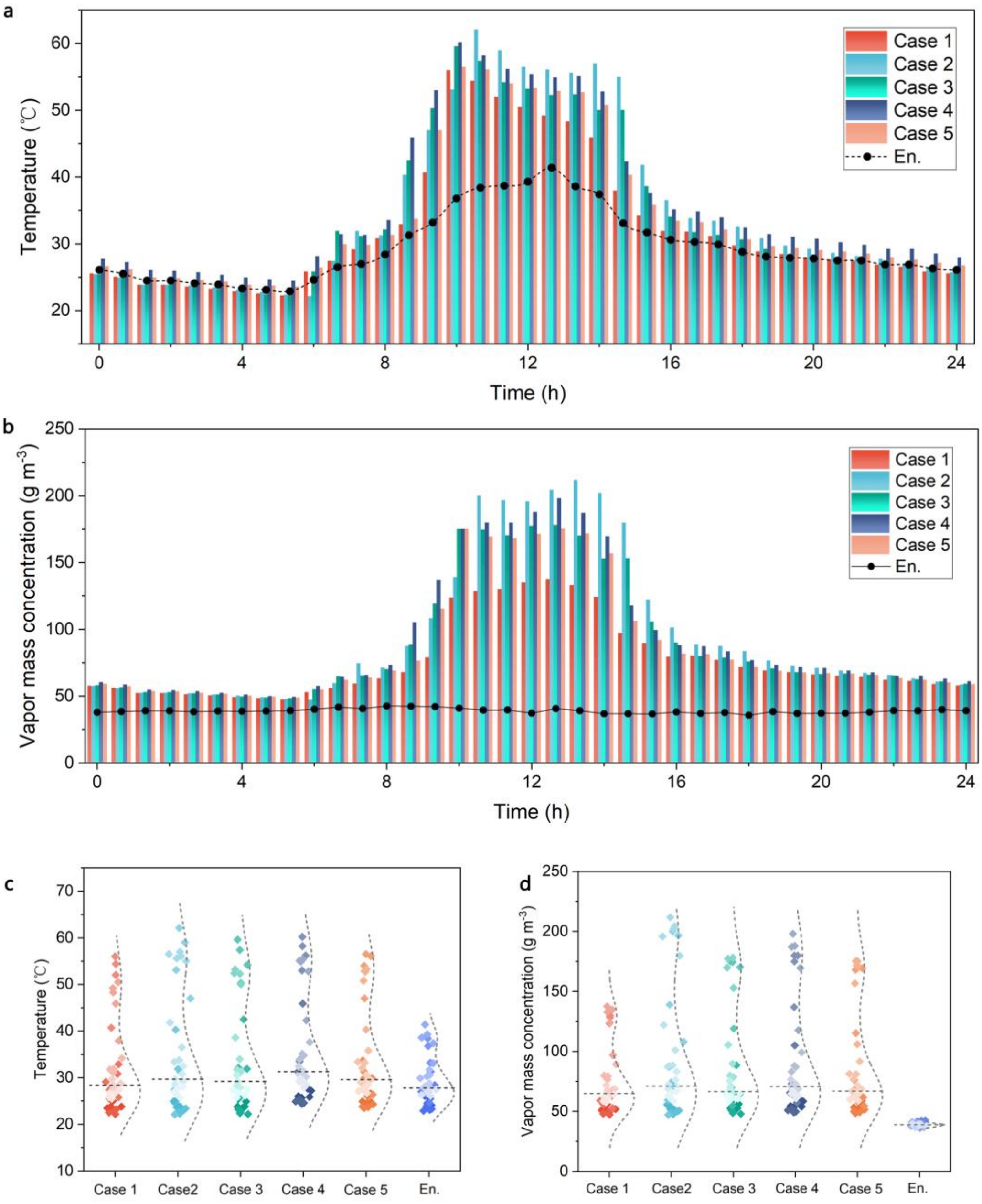

Supplementary Fig. 6 Operation parameters in cases 1–5 and of the environment. (a) Daily temperature changes vs. time. (b) Daily water vapor concentration changes vs. time. Statistical distribution of the (c) temperature and (d) water vapor concentration. The shades of the color from deep to light depicts the 0 to 24 h, respectively. Case 1 is a reference system without solar evaporators. Case 2 includes solar evaporators. Case 3 further pumps vapor out through a condensing tube for forced condensation with additional photovoltaics. Case 4 uses coated glass (condensation-enhanced) to condense the water without external energy input. Case 5 integrates both the condensing tube (powered by photovoltaics) and condensation-enhanced glass for condensation. En. represents the environmental parameters.

Supplementary Note 2. The dependence of solar-water energy efficiency

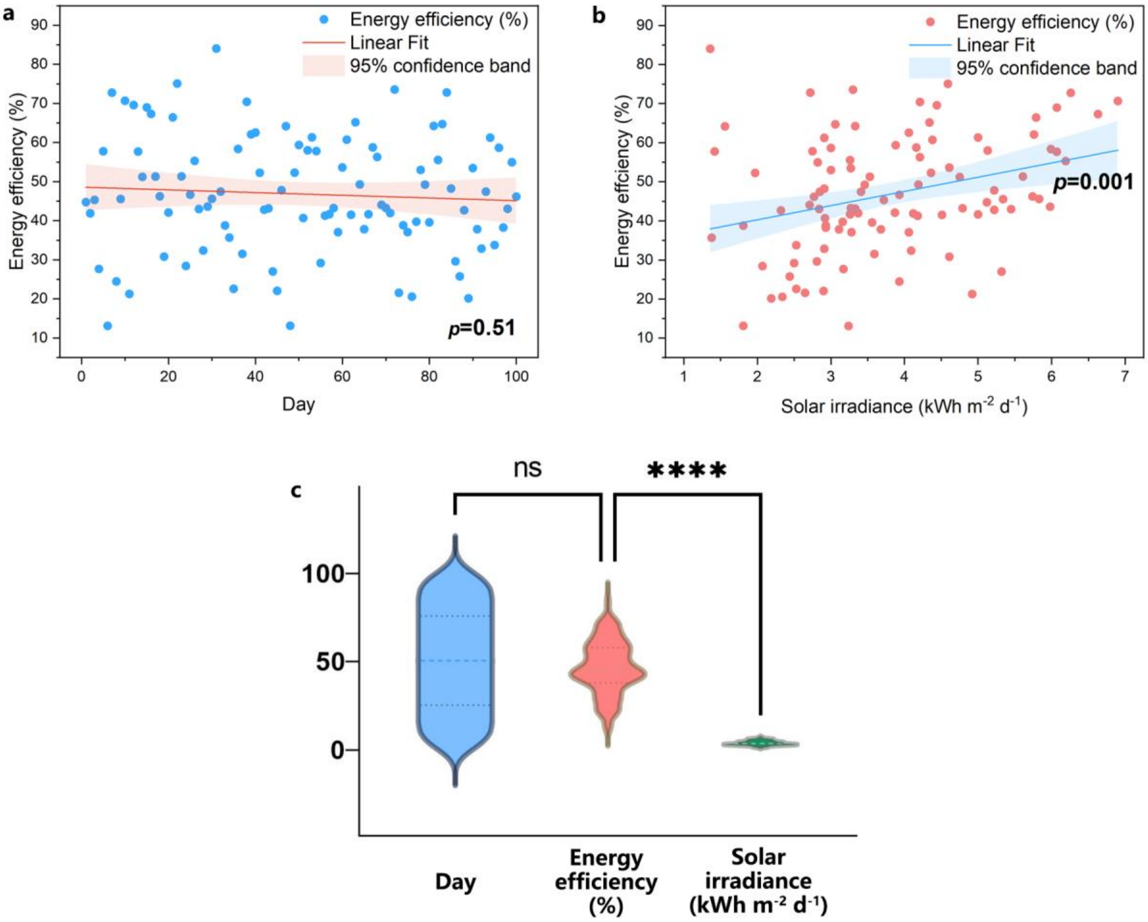

Supplementary Fig. 7 The linear regression between solar-water energy efficiency and (a) Day (Slope=-0.03, p=0.51, ns), (b) Solar irradiance (Slope=3.6, p=0.001, ***). (c) Between-group comparisons of the day, energy efficiency, and solar irradiance (Day vs. Energy efficiency, p>0.05, ns; Energy efficiency vs. Solar irradiance, p<0.0001, ****).

By linear regression of energy efficiency and the day (Supplementary Fig. 7a), the p-value of the slope is 0.51, demonstrating that the linear relationship between them is not significant. In contrast, the p-value of the slope between the energy efficiency and the solar irradiance is 0.001, demonstrating a significant linear relationship between them (Supplementary Fig. 7b).

Moreover, between-group comparisons (Day, Energy efficiency, and Solar irradiance) were performed using independent samples Kruskal-Wallis one-way analysis of variance (ANOVA). The results showed that the correlation between the energy efficiency and the day is not significant

($p>0.9999$) while the correlation between the energy efficiency and the solar irradiance is significant ($p<0.0001$, Supplementary Fig. 7c).

Supplementary Table 1 The Spearman coefficients between solar-water energy efficiency and Day or Solar irradiance

Spearman coefficients	Day	Solar irradiance
Solar-water energy efficiency	-0.078	0.34
p-value	0.44	4.7×10^{-4}

Correlation analysis was further implemented to test for the effects of day and solar irradiance on solar-water energy efficiency. The data regarding solar irradiance and solar-water energy efficiency were ln transformed before analysis to minimize the impacts of the outliers. The results show that the solar-water energy efficiency has no significant relation with the pilot experiment day with $p>0.05$. On the contrary, the Spearman coefficient is 0.34 between the efficiency and the solar irradiance with $p<0.001$.

From the analysis above, it is concluded that solar-to-water energy efficiency is positively related to solar irradiance while it stays stable within the 100-day operation.

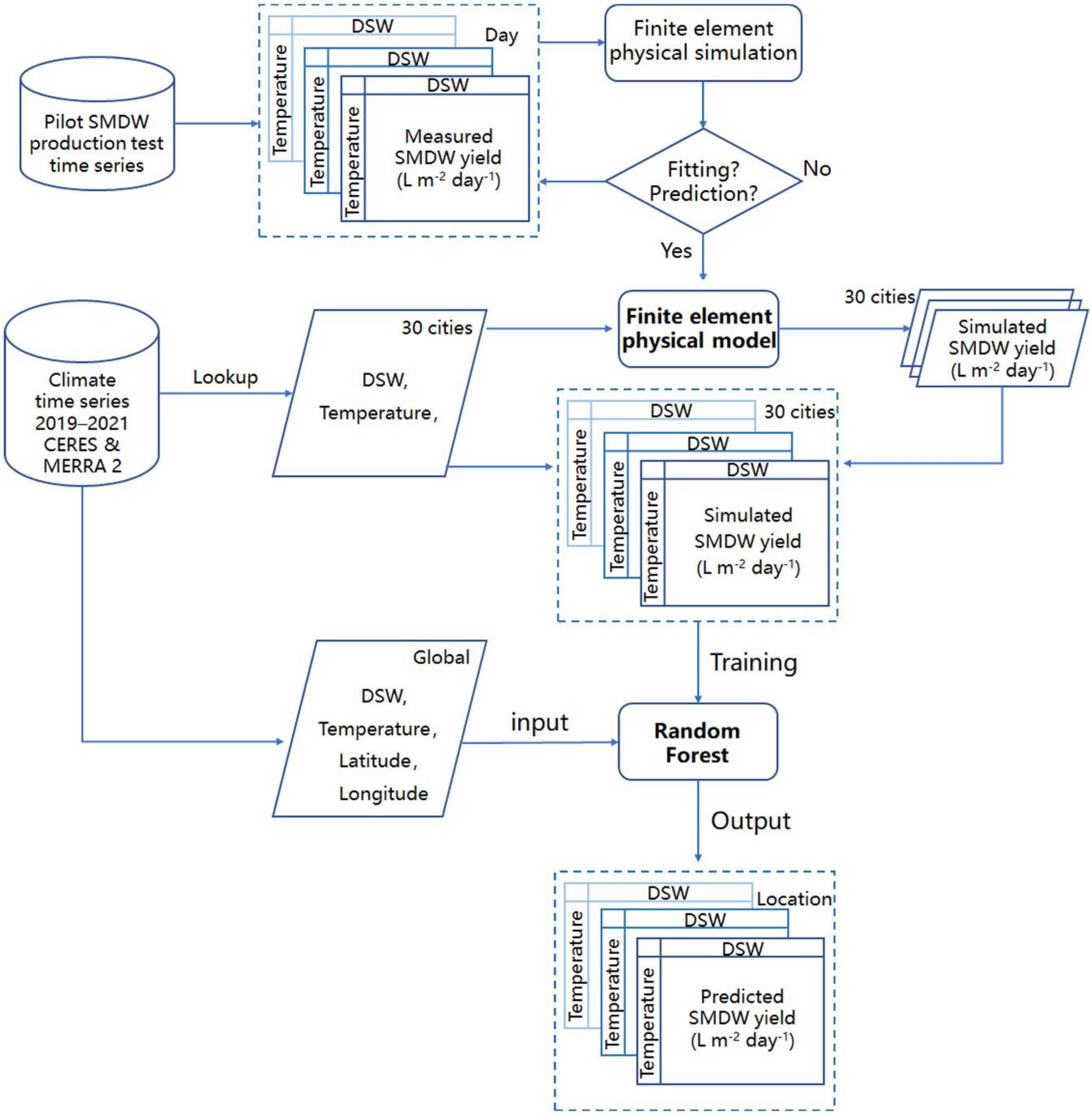

Supplementary Fig. 8 Data processing workflow of the physics-guided machine learning model to predict the safely managed drinking water (SMDW). Cylinders indicate the data from the pilot study, Clouds and the Earth's Radiant Energy System (CERES) and Modern-Era Retrospective analysis for Research and Applications, Version 2 (MERRA 2). The rest are table frames (the output documents), rhombus (judgments of the model), rectangles (data processing), and parallelograms (output datasets). Downward shortwave irradiation, DSW.

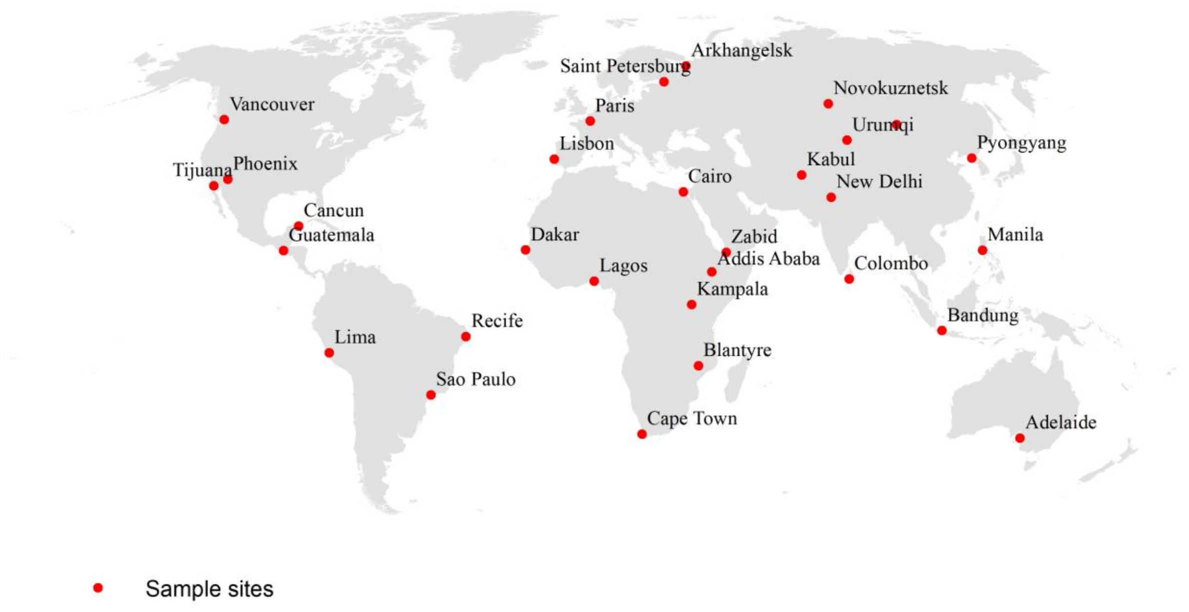

Supplementary Fig. 9 The selected sites for the training sets of the physics-guided machine learning model.

Supplementary Table 2. Locations of the selected 30 cities over the world

City	Longitude	Latitude
Tijuana	-115.7439	31.7774
Lima	-76.4472	-12.3607
Sao Paulo	-46.5646	-23.5229
Recife	-35.0949	-8.0802
Guatemala	-90.4729	14.5922
Cancun	-86.8254	21.0774
Lagos	3.4156	6.5736
Dakar	-17.3282	14.7607
Cairo	31.3193	30.0944
Addis Ababa	38.7241	9.0008
Zabid	43.3325	14.1623
Kampala	32.5934	0.3286
Blantyre	34.9884	-15.846
Cape Town	18.6595	-33.904
Adelaide	138.7666	-34.9316
Kabul	69.377	34.5693

New Delhi	77.1993	28.6996
Colombo	79.9441	7.1402
Bandung	107.7573	-6.5379
Manila	120.809	14.6857
Urumqi	87.5864	43.8195
Ulaanbaatar	106.8674	48.0789
Saint Petersburg	30.4245	59.9336
Arkhangelsk	40.488	64.5961
Novokuznetsk	87.1117	53.7366
Pyongyang	125.8023	39.0779
Lisbon	-9.1743	38.766
Phoenix	-112.0436	33.4795
Vancouver	-123.1398	49.3626
Paris	2.5218	48.9748

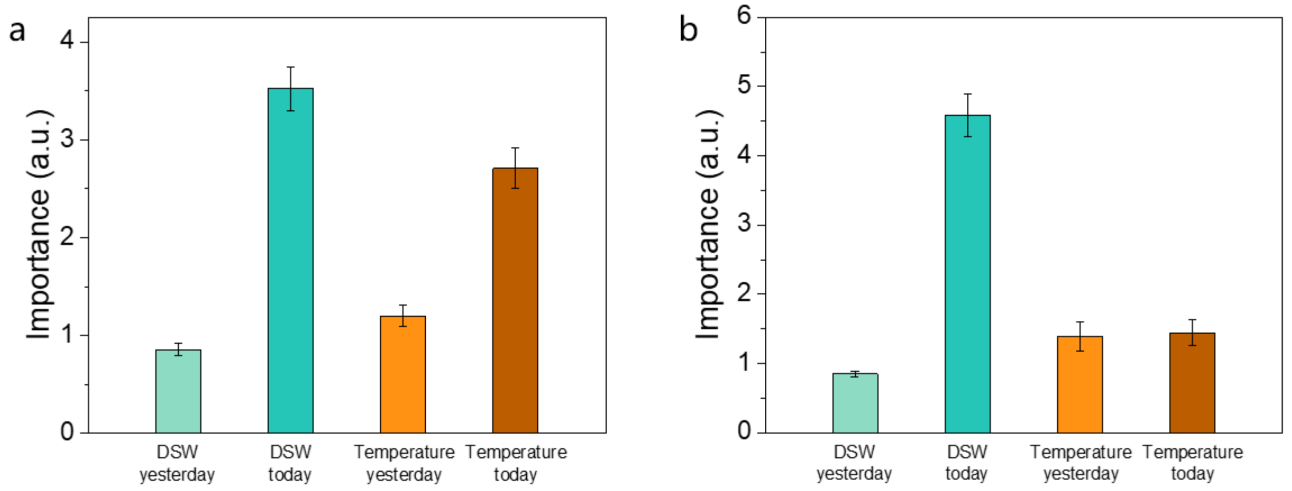

Supplementary Fig. 10 The importance of each input parameter in the physics-guided machine learning model for (a) evaporation-optimized case (Eva. opt.) and (b) evaporation-condensation-optimized case (Eva.-cond. opt.). For each variable, the rise in prediction error if the values of each variable are permuted across the out-of-bag observations was measured. This measure is computed for every tree, then averaged over the entire ensemble and divided by the standard deviation over the entire ensemble. Downward shortwave irradiation, DSW.

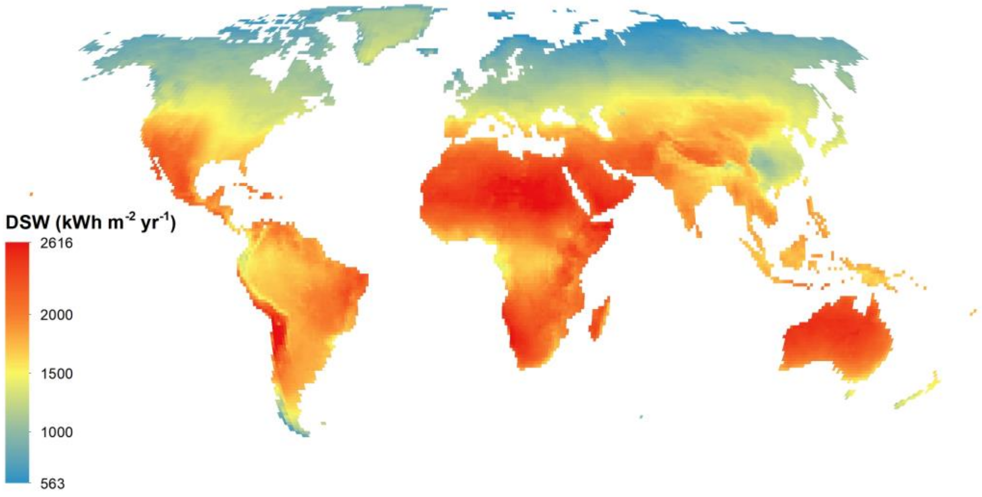

Supplementary Fig. 11 Global distribution of the yearly total surface downward shortwave irradiation (DSW).

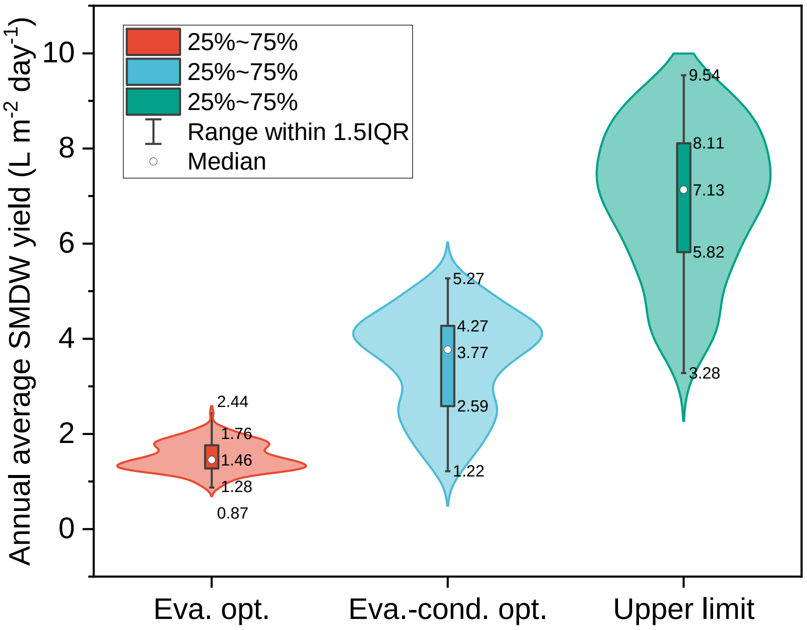

Supplementary Fig. 12 The violin plot of annual average safely managed drinking water (SMDW) yield under the three scenarios. Interquartile Range, IQR; Evaporation-optimized case, Eva. opt.; evaporation-condensation-optimized case, Eva.-cond. opt. The upper limit refers to the scenario that converts all the sunlight to power the solar evaporation for SMDW yield.

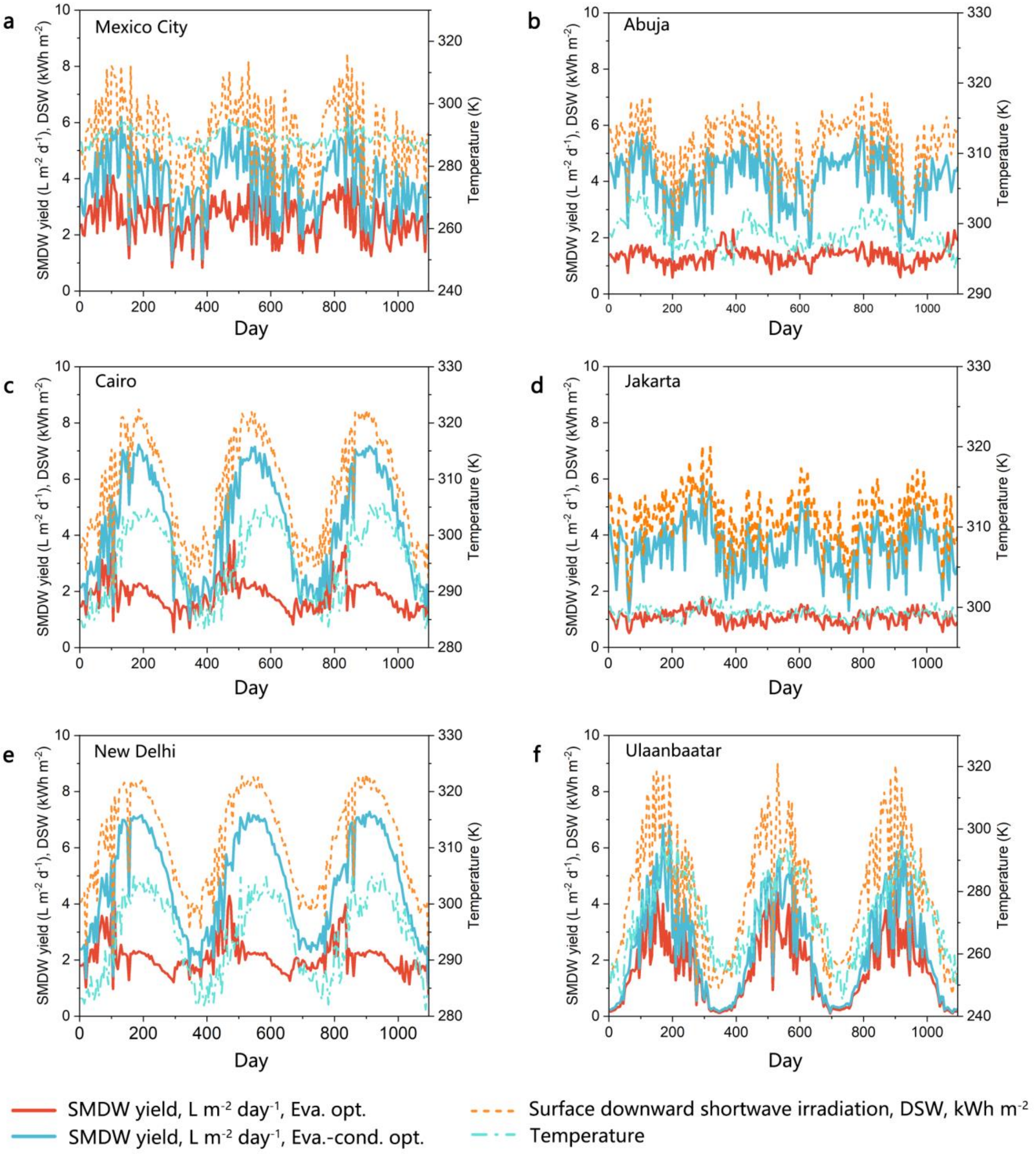

Supplementary Fig. 13 Seasonal variations of the safely managed drinking water (SMDW) yield for 6 six representative cities across the world. (a) Mexico City. (b) Abuja. (c) Cairo. (d) Jakarta. (e) New Delhi. (f) Ulaanbaatar. Evaporation-optimized case, Eva. opt.; evaporation-condensation-optimized case, Eva.-cond. opt..

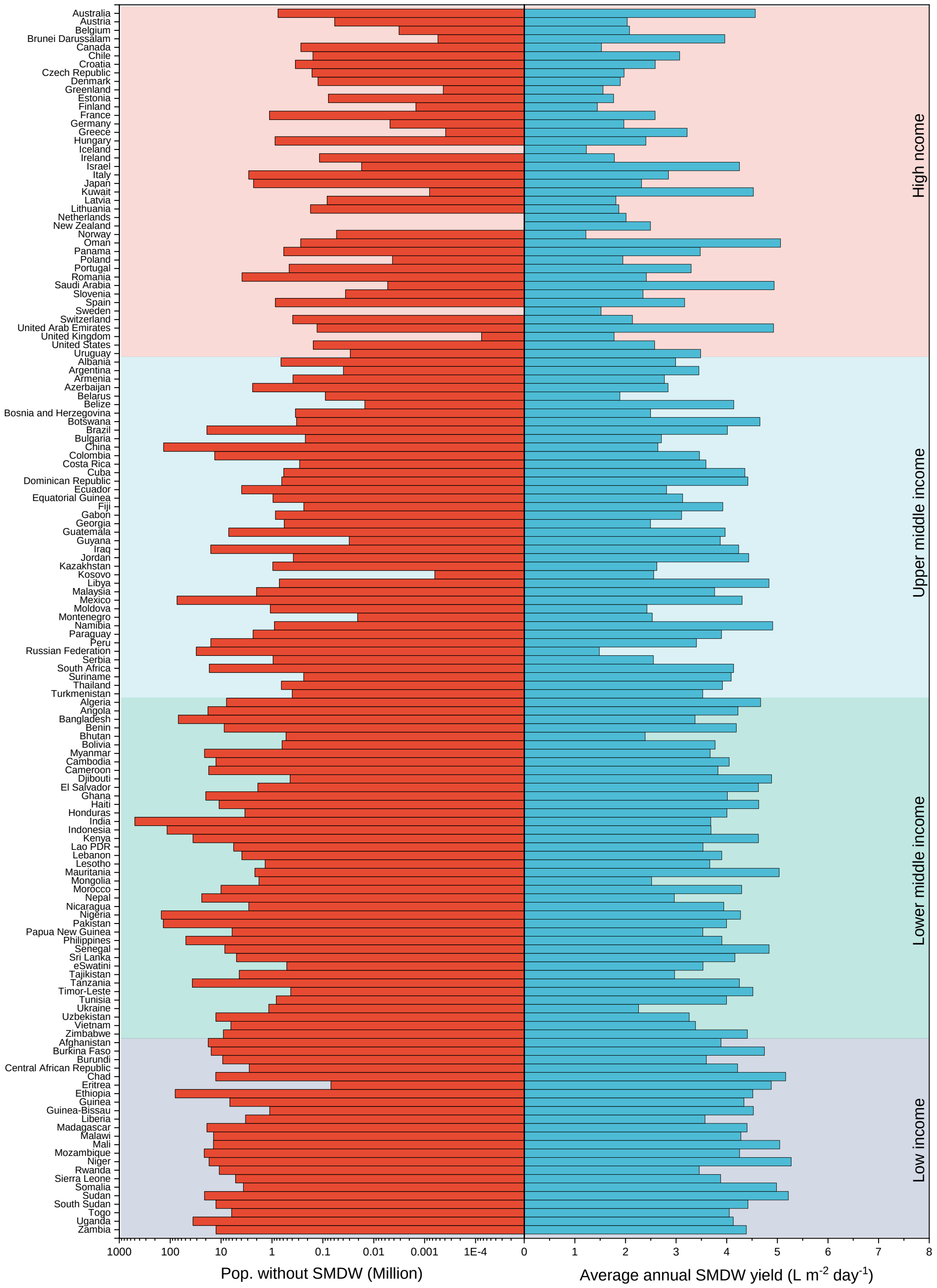

Supplementary Fig. 14 Pyramid chart of country data on average annual water production and water-scarce population (evaporation-condensation-optimized system). The low income, lower middle income, upper middle income and, upper income countries are classified by the World Bank (https://blogs.worldbank.org/en/opendata/new-world-bank-country-classifications-income-level-2022-2023).

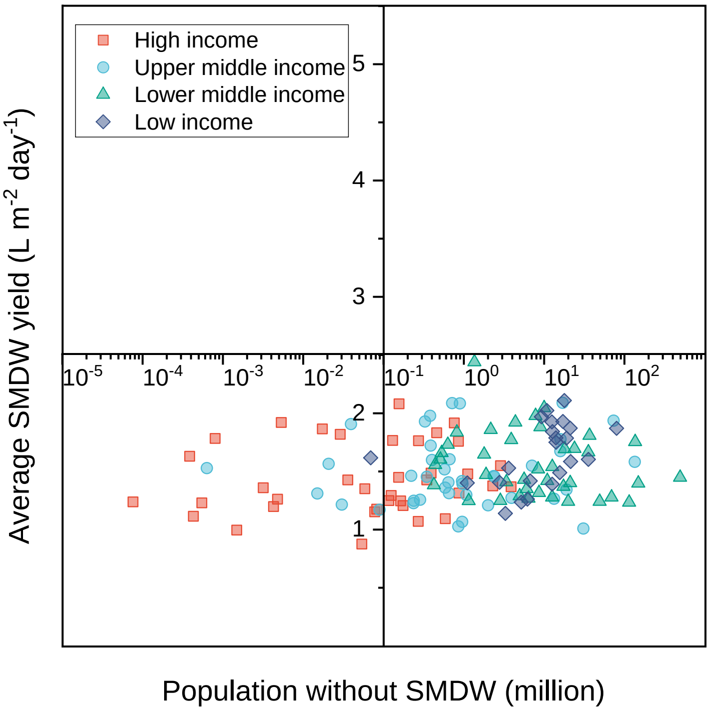

Supplementary Fig. 15 Four quadrant charts of the annual safely managed drinking water (SMDW) yield (evaporation-optimized system) concerning the population without SMDW of different income-level countries. The low income, lower middle income, upper middle income and, upper income countries are classified by the World Bank (https://blogs.worldbank.org/en/opendata/new-world-bank-country-classifications-income-level-2022-2023).

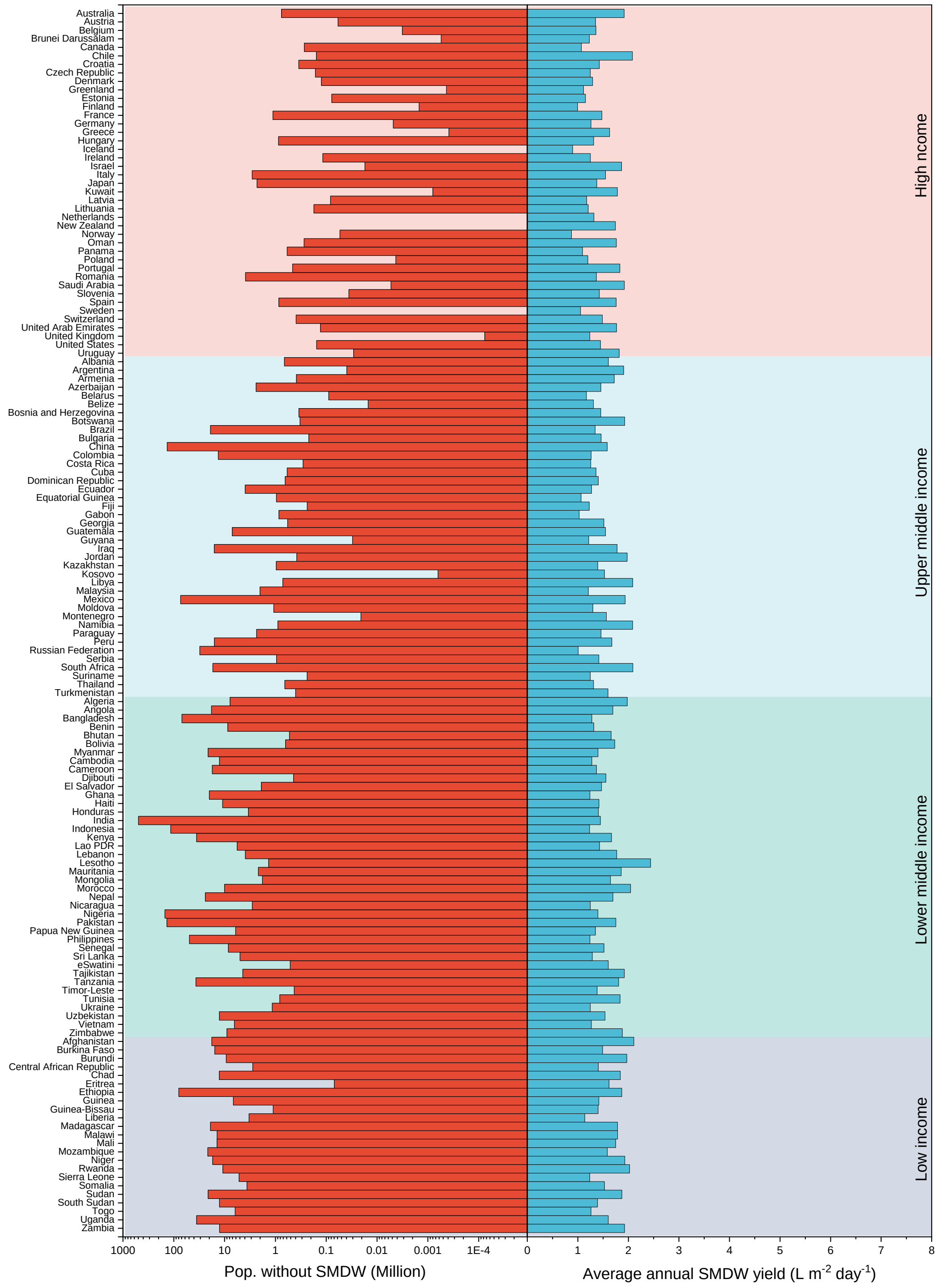

Supplementary Fig. 16 Pyramid chart of country data on average annual safely managed drinking water (SMDW) production and water-scarce population (evaporation-optimized system). The low income, lower middle income, upper middle income and, upper income countries are classified by the World Bank (https://blogs.worldbank.org/en/opendata/new-world-bank-country-classifications-income-level-2022-2023).

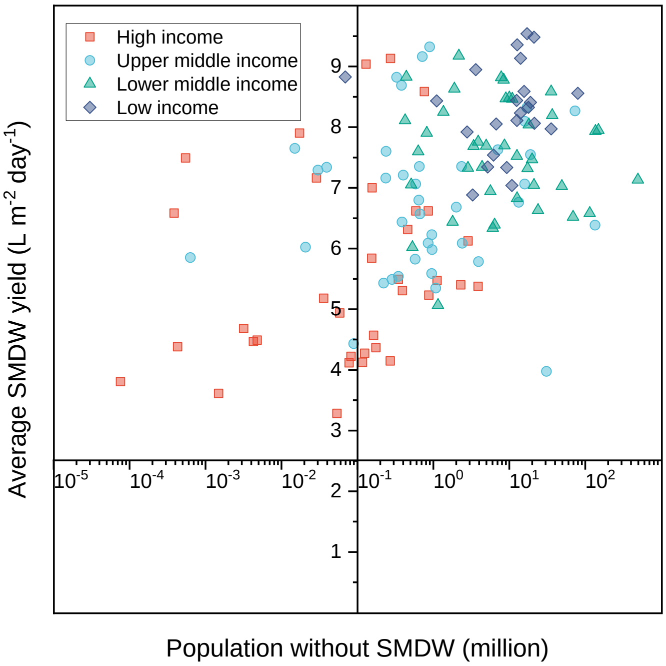

Supplementary Fig. 17 Four quadrant charts of the annual safely managed drinking water (SMDW) yield (Upper limit) concerning the population without SMDW of different income-level countries. The low income, lower middle income, upper middle income and, upper income countries are classified by the World Bank (https://blogs.worldbank.org/en/opendata/new-world-bank-country-classifications-income-level-2022-2023).

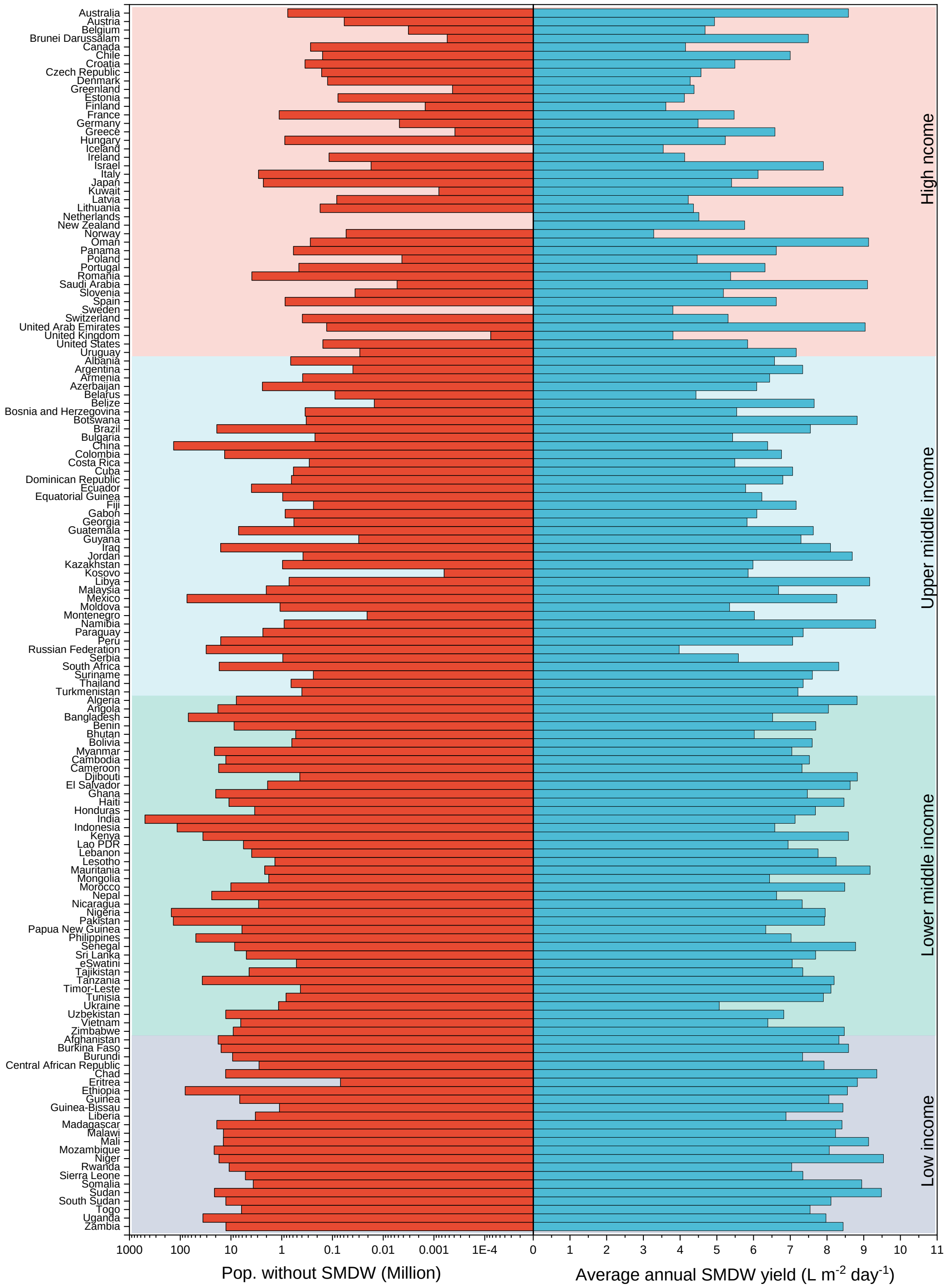

Supplementary Fig. 18 Pyramid chart of country data on average annual safely managed drinking water (SMDW) production and water-scarce population (upper limit). The low income, lower middle income, upper middle income and, upper income countries are classified by the World Bank (https://blogs.worldbank.org/en/opendata/new-world-bank-country-classifications-income-level-2022-2023).

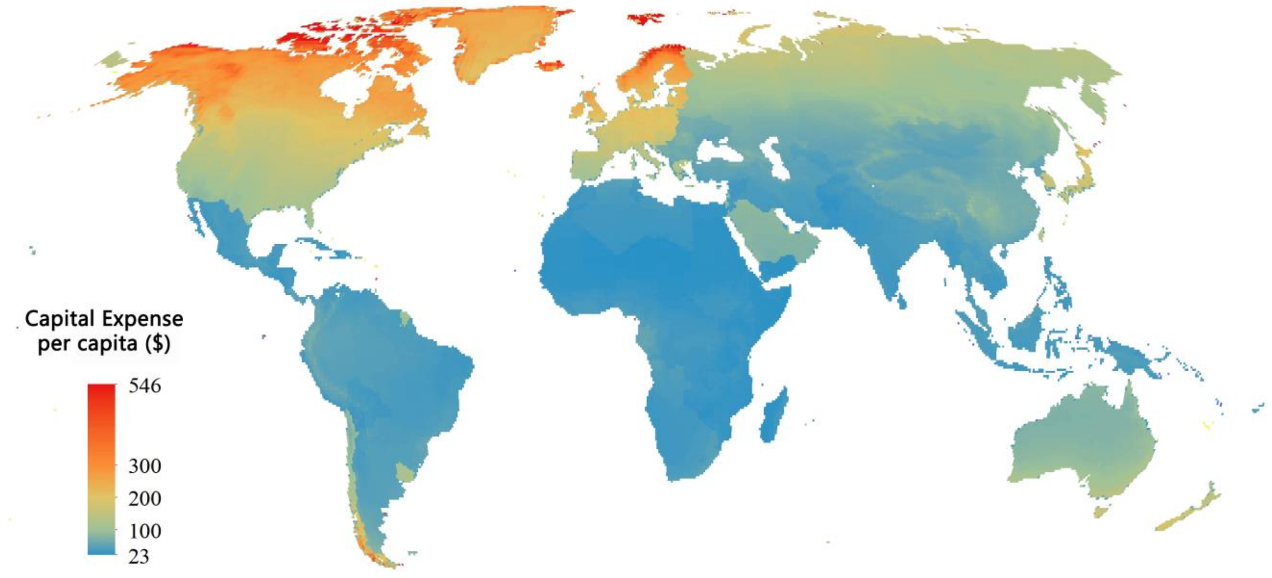

Supplementary Fig. 19 Map of the Capital expense per capita of solar water evaporation (evaporation-condensation-optimized system). The calculation of capital expense can be seen in the supplementary note 3.

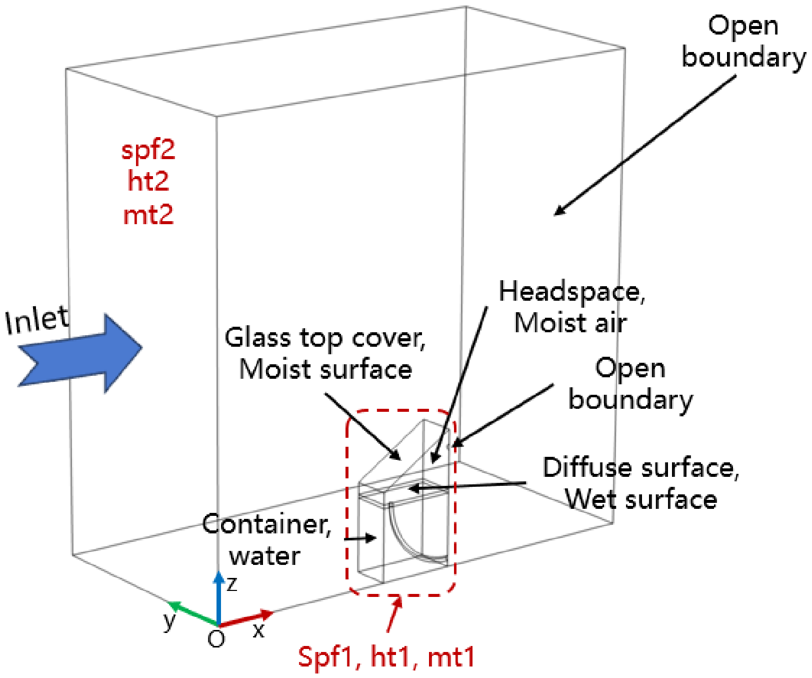

Supplementary Fig. 20 Schematic diagrams of the COMSOL model setups. COMSOL refers to the software "COMSOL Multiphysics". Moist surface refers to the condensation surface. The wet surface refers to the evaporation surface. Open boundary on the container surface refers to the boundary that connects the inner space of the container and outer atmosphere. The open boundary of the atmosphere refers to the outlet of the wind. Single-phase flow (spf), heat transfer (ht), moist transfer (mt) and radiation (rad) are modules in the COMSOL Multiphysics, and 1 refers to the domain in the solar evaporation device, and 2 refers to the atmosphere domain.

Supplementary Note 3. Cost estimation

Capital cost:

Supplementary Table 3 The capital cost per area

Classification	Content	Unit price	Usage per area	Cost per area/$ m^{-2}
Raw materials	Solar evaporator	$0.070 kg^{-1}	6.0 kg m^{-2}	0.417
	Sodium Alginate	$3.0 kg^{-1}	0.0008 kg m^{-2}	0.024

	PVA	\$1.3 kg^{-1}	0.0008 kg m^{-2}	0.0104
	Poly(methyl methacrylate)	\$2.2 kg^{-1}	18.17 kg m^{-2}	40.44
	Glass	\$0.24 kg^{-1}	14.43 kg m^{-2}	3.44
Energy	Electricity	\$0.088 kWh^{-1}	3 kWh m^{-2}	0.265
Facilities	Water container	-	\$1 m^{-2}	1.0
	Silicone tubes	\$2.55 kg^{-1}	0.063 kg m^{-2}	0.161
Total				45.7574

The raw material of the solar evaporator is sugarcane and the cost is its price. The usage of the sugarcane (kg m^{-2}) is estimated assuming that sugarcane has a density of water (1g cm^{-3}) as 1/(1000g/1g cm^{-3}/0.6 cm)*10000 cm^2 m^{-2}, where 0.6 cm is the thickness of the solar evaporator (sugarcane).

Sodium alginate (SA), PVA usage was calculated as 200 mL m^{-2} * 0.04 g/100 mL/1000 g kg^{-1}, 200 mL m^{-2} is the amount of the cast solution used per square meter and 0.04 g/100 mL is the concentration of the SA and PVA solutions.

Poly(methyl methacrylate) usage (kg m^{-2}) was calculated considering a device with a floor area of 4 m^2, container height of 0.2 m, top cover tilting angle of 30°: (2 m*2 m+0.2 m*2 m+(0.2 m+1.36 m)*2 m/2*2+1.36 m*2)* 0.006 m*1.19*10^3 kg m^{-3}/4 m^2, where 2 m is the bottom side length, 1.36 m is the backboard height, 0.006 m is the thickness of the poly(methyl methacrylate) plate and 1.18*10^{-3} kg m^{-3} is the density.

The glass usage (kg m^{-2}) is calculated considering a device with a floor area of 4 m^2, container height of 0.2 m, top cover tilting angle of 30°: 2.32 m*2 m* 0.005 m*2500 kg m^{-3}/4 m^2, where 0.005 m is the thickness and 2500 kg m^{-3} is the density of glass.

The cost of electricity is estimated according to the manufacturing energy of devices, including solar evaporators and coating fabrication. The cost of facilities mainly includes water containers and silicone tubes.

Supplementary Table 4 Labor cost estimation

National income levels	Median monthly wage/$	Labor costs in terms of wages
Low income	106.23	5.68
Lower-middle income	193.41	10.35
Upper-middle income	390.00	20.86
High income	2075.06	111.00

The low income, lower middle income, upper middle income and, upper income countries are classified by the World Bank (https://blogs.worldbank.org/en/opendata/new-world-bank-country-classifications-income-level-2022-2023). We take the median monthly income of China as a reference and estimate the labor cost to be \$20.86 m^{-2} according to the actual manufacturing price for the solar evaporation devices. Then the labor cost is categorized into "low-income", "lower-middle income", "upper-middle income" and "high-income" countries. The labor costs were obtained by normalizing the median monthly wage of these four categories of countries.

Operation and maintenance cost:

Our devices mainly comprise acrylic containers, carbon solar evaporators, top cover glass, hydrogel-based condensation coatings, and connecting silicone tubes.

The **operation cost** refers to the total price of the parts in the solar evaporation device that need to be replaced after a certain period. It mainly includes solar evaporators and hydrogel-based condensation coatings.

The solar absorber used in our study is inorganic biochar, which is stable in the environment. According to previous reports, solar stills composed of typical inorganic carbon-based black paint exhibit a long lifespan ranging from 2-10 years[1-3]. In addition, during our 100-day outdoor test, the solar absorbers showed no observable deterioration. Thus, its lifespan is estimated at 2 years.

The coating for accelerated condensation is composed mainly of PVA fibers. PVA is chemically stable and widely used in coatings and fibers. As previously reported, PVA coatings are stable (remain hydrophilic and antifogging) after 2-7 months of exposure to a hot and humid environment or daily

use[4,5]. Moreover, PVA has a strong bond with the matrix, exhibiting resistance to an alkaline environment. They also estimated that the tensile strength of PVA fiber could be preserved after even 60 years of ultraviolet irradiance in a hot environment[6]. In addition, the coating used in our outdoor test shows no obvious deterioration after 100 days, so its lifespan is also estimated at 2 years for simplification of calculations.

Therefore, from Supplementary Table 3, the solar evaporator, sodium alginate, PVA, and the electricity used for fabrication is a total of \$0.72 m^{-2}, which is considered as consumable material and should be replaced. The operation cost was comprised of the substitution of the solar evaporators and condensation coatings and the auxiliary software cost (2% of the materials cost).

The **maintenance cost** refers to the repair of the whole solar evaporation device (whole capital cost). For the whole devices mainly made of Poly(methyl methacrylate) (PMMA), its lifetime is estimated as 10 years considering the property of the PMMA[7,8]. Therefore, besides the operation cost to replace the solar evaporators and the condensation coatings, the maintenance costs were estimated at 3% of the capital costs per year and comprised 30% of the capital costs to fix the problems that the whole solar evaporation device may encounter.

Supplementary References:

1 Ni, G. *et al.* A salt-rejecting floating solar still for low-cost desalination. *Energy Environ. Sci.* **11**, 1510-1519 (2018).

2 Kabeel, A. E. *et al.* Effect of water depth on a novel absorber plate of pyramid solar still coated with TiO_2 nano black paint. *J. Cleaner Prod.* **213**, 185-191 (2019).

3 Abdullah, A. S., Younes, M. M., Omara, Z. M. & Essa, F. A. New design of trays solar still with enhanced evaporation methods – Comprehensive study. *Sol. Energy* **203**, 164-174 (2020).

4 Yang, M. *et al.* Structure and properties of polyvinyl alcohol (PVA)/Al_2O_3 antifogging coating with self-healing performance. *J. Coat. Technol. Res.* (2024).

5 Yu, X. *et al.* Highly durable antifogging coatings resistant to long-term airborne pollution and intensive UV irradiation. *Mater. Des.* **194** (2020).

6 Silva, F. A., Peled, A., Zukowski, B. & Toledo Filho, R. D. in *A Framework for Durability Design with Strain-Hardening Cement-Based Composites (SHCC): State-of-the-Art Report of the RILEM Technical Committee 240-FDS* (eds Gideon P. A. G. van Zijl & Volker Slowik) 59-78 (Springer Netherlands, 2017).

7 Halliwell, S. M. *Weathering of plastics glazing materials*, Loughborough University of Technology, (1996).

8 Gilbert, M. in *Brydson's Plastics Materials (Eighth Edition)* (ed Marianne Gilbert) 75-102 (Butterworth-Heinemann, 2017).
